# Rapid Characterization of Vegetation Structure with a Microsoft Kinect Sensor

**DOI:** 10.3390/s130202384

**Published:** 2013-02-11

**Authors:** George Azzari, Michael L. Goulden, Radu B. Rusu

**Affiliations:** 1 Department of Earth System Science, University of California Irvine, Irvine, CA 92697, USA; E-Mail: mgoulden@uci.edu; 2 Open Perception, Inc., 68 Willow Road, Menlo Park, CA 94025, USA; E-Mail: rusu@openperception.org

**Keywords:** terrestrial ecology, field measurements, canopy structure, biomass, LIDAR, Microsoft Kinect, point clouds, depth images, convex hulls, concave hulls

## Abstract

The importance of vegetation structure and biomass in controlling land-atmosphere exchange is widely recognized, but measurements of canopy structure are challenging, time consuming, and often rely on destructive methods. The Microsoft Kinect is an infrared sensor designed for video gaming that outputs synchronized color and depth images and that has the potential to allow rapid characterization of vegetation structure. We compared depth images from a Kinect sensor with manual measurements of plant structure and size for two species growing in a California grassland. The depth images agreed well with the horizontal and vertical measurements of plant size made manually. Similarly, the plant volumes calculated with a three-dimensional convex hulls approach was well related to plant biomass. The Kinect showed some limitations for ecological observation associated with a short measurement range and daytime light contamination. Nonetheless, the Kinect's light weight, fast acquisition time, low power requirement, and cost make it a promising tool for rapid field surveys of canopy structure, especially in small-statured vegetation.

## 1. Introduction

The importance of vegetation structure and biomass in controlling terrestrial ecosystem function and land-atmosphere exchange is widely recognized. Canopy architecture affects the interception of light by leaves, which is a dominant factor controlling primary production, evapotranspiration, and plant competition [1]. Vegetation structure influences ecosystem-atmosphere mass and energy exchange through variation in albedo, emissivity, latent heat flux, and sensible heat flux; these properties ultimately affect climate on local and regional scales [2–5]. The accumulation of biomass plays a major role in the local and global carbon cycle, while also determining fuel accumulation and contributing to soil nutrient balance [6].

Canopy light transfer models, which are basic components of land surface models, often rely on simplified canopies that represent plants as solid shapes such as boxes, spheres, cylinders, or cones ([1], and references therein). Biomass is often estimated using allometric relations based on similar simplified assumptions about plant shape and volume. Realistic information on stand structure, especially if extended in space and time, could improve biophysical canopy models and biomass estimations, while contributing to our understanding of ecosystem function.

Measurements of canopy structure are challenging [6]. Remotely sensed measurements from satellites can provide large scale data with good spatial and temporal resolution [6–8], but they require validation on the ground. Ground-based measurements are costly and time consuming, and often rely on destructive methods. Field data are usually collected over small areas; it is difficult to scale up these observations to longer time periods or larger spatial scales. Laser scanning systems such as LIDAR have recently become more available, offering an effective non-destructive method for airborne and ground measurements [9–15]. However, LIDAR remains expensive and data availability is still limited, especially time-series of canopy structure at individual locations. Inexpensive and portable instrumentation for ground-based measurements of biomass and vegetation structure would increase the spatial and temporal coverage of structural measurements, and might be integrated into large instrument networks such as FluxNet or SpecNet [16–18].

The consumer market for video-games and digital entertainment has expanded dramatically in the last three decades, bringing the cost of advanced technology to affordable levels. The Kinect sensor produced by Microsoft is a good example of this technology: Kinect is an infrared sensor designed to track body position and movement at a single-articulation level. Kinect sensors are available for US $150, which corresponds to roughly 0.1% of the cost of a research-grade, ground-based LIDAR system. In this “proof of concept” paper, we show that the Kinect sensor is useful for field measurements of vegetation structure, including base diameter, height, and volume, and for assessing the optimal solid shape approximation for canopy modelling and biomass estimation. We made observations on 4 to 8 replicates of two plant species to test the performance of Kinect sensors in the field. We directly compared measurements of basal diameter and height taken manually with those derived from vegetation point clouds acquired with a Kinect. We also compared the plant volumes calculated with point-cloud-derived convex hulls against alternative solid shape approximations. Finally, we measured the dry biomass of the sample plants, and used the Kinect-derived structural variables to derive allometric relations.

## 2. Methods

### 2.1. Microsoft Kinect Sensor

Kinect captures synchronized color and depth images at a rate of 30 frames per second (*fps*) and with a field of view of 57° × 43°, using a RGB camera (8 *bit* VGA resolution with 640 × 480 *pixel*) aligned with a depth imager. The Kinect depth sensor was designed by PrimeSense, and is composed of an IR laser projector and a monochrome 640 × 480 *pixel* IR CMOS sensor. Depth information is output with 11 *bit* precision for each pixel [19]. Kinect's depth measurement principle differs from laser scanners such as LIDAR. LIDAR produces depth images by measuring the time of flight of individual laser pulses that sequentially scan the entire scene using motorized pan-tilt units. Kinect's laser projector illuminates the entire scene at once, using a diffraction grid that imposes a consistent pattern of speckles on the beam [20]. Depth is then calculated by correlation and triangulation between the laser pattern captured by the CMOS sensor and a reference pattern stored in the sensor's memory [20,21]. This acquisition method bypasses the need for moving parts, which reduces the sensor's weight, size, acquisition time, and power requirement.

Using external software, depth images can be converted and stored in three-dimensional representations called point clouds. Each point in the cloud is represented by a set of coordinates *P* = {*x*, *y*, *z*}, which defines its position in space. Point coordinates can also be extended to the form *P* = {*x*, *y*, *z*, *R*, *G*, *B*}, which includes the red, green, and blue components recorded by the aligned RGB camera, or *P* = {*x*, *y*, *z*, *I*}, which includes intensity information. Only point clouds with *x*, *y*, *z* coordinates were used in this study. Kinect-derived point clouds, which contain about 300,000 points for each frame, have a 3 *mm* error along the horizontal axes. Error increases with distance from the sensor [20]; the depth error is ±1 *cm* at 2 *m* and ±7 *cm* at 5 *m* [20]. The optimal measurement range recommended by Microsoft is 1.2–3.5 *m*, though tests show Kinect is capable of measurements at 0.8–6.0 *m* [19]. Additional factors influence depth errors, including light conditions and target reflectivity. Bright light and high reflectivity reduce the laser pattern contrast on targeted surfaces, which creates gaps and outliers in the resulting point cloud [20], as also shown in our preliminary tests (see Section 3.1).

### 2.2. Acquisition, Processing and Analysis of Vegetation Point Clouds

Acquisition, processing and analysis of point clouds were performed using tools and methods included in an open-source C++ library called Point Cloud Library (PCL, [22]). A detailed description of these methods is beyond the scope of this paper; full documentation is provided on the PCL website (www.pointclouds.org).

#### 2.2.1. Point Clouds Acquisition

**Figure 1.**
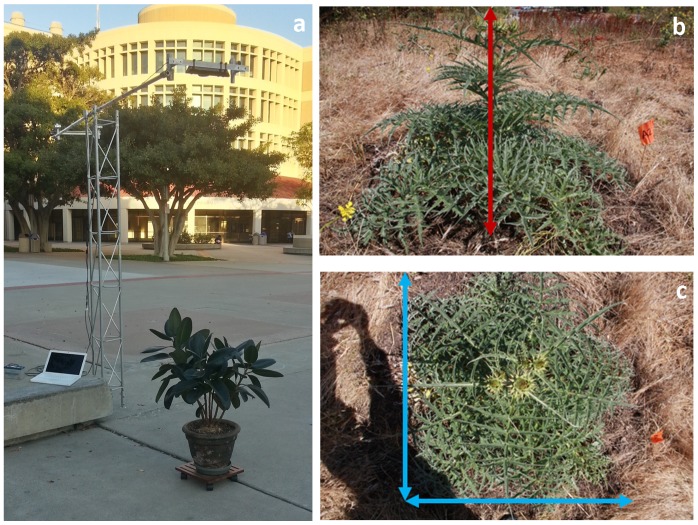
(**a**) The “tower mode” experimental setup used for nadir measurements with Kinect (see Sections 2.3 and 2.4). (**b**) Side view and (**c**) nadir view of a wild artichoke plant recorded during the field test (see Section 2.4). Blue and red lines represent plant's basal diameter and height as measured manually with a ruler.

Point clouds were acquired from Kinect using the PCL OpenNI grabber, an I/O interface that is compatible with many devices. The acquisition software ran on a laptop computer with Linux Ubuntu 12.04 LTS. Two acquisition strategies were used in this paper: (*i*) “Tower Mode”: The Kinect was mounted on a 1.4 *m* long mast extending horizontally from a 3 *m* tall aluminum tower. The sensor was oriented to provide a nadir view of a plant (see Figure 1(a)). Acquisition from the tower setup was operated in single-shot mode: a single point cloud was recorded along with the corresponding raw infrared image. (*ii*) “Multi-angular Mode”: The Kinect was manually moved 360 degrees around a plant, while a stream of point clouds was recorded. A complete 360 degrees scan of a plant yielded about 2000 point clouds and took up about 2 GB of disk space. Streamed multi-angular point clouds were then merged into a single cloud using the Kinfu PCL utility (Kinect Fusion, [23]). Kinfu does not rely on targets or user interaction to co-register point clouds. Because of its high computational needs, Kinfu must be run on computers equipped with Graphic Processors Units (GPU). If the acquisition machine is equipped with such a device, the co-registration can be performed in real time, rather than in “offline” mode on recorded streams.

#### 2.2.2. Canopy Structure from Point Clouds

The point clouds were then processed following four steps: (i) Noise reduction and filtering to remove outliers, (ii) Delineation of individual plants and point extraction, (iii) Calculation of plant *x*, *y*, and *z* size, and (iv) Calculation of plant volume. All the post-processing and analysis were performed in the lab using a desktop computer equipped with a 1.5 Gb GPU running Linux Ubuntu 12.04 LTS.

**Figure 2.**
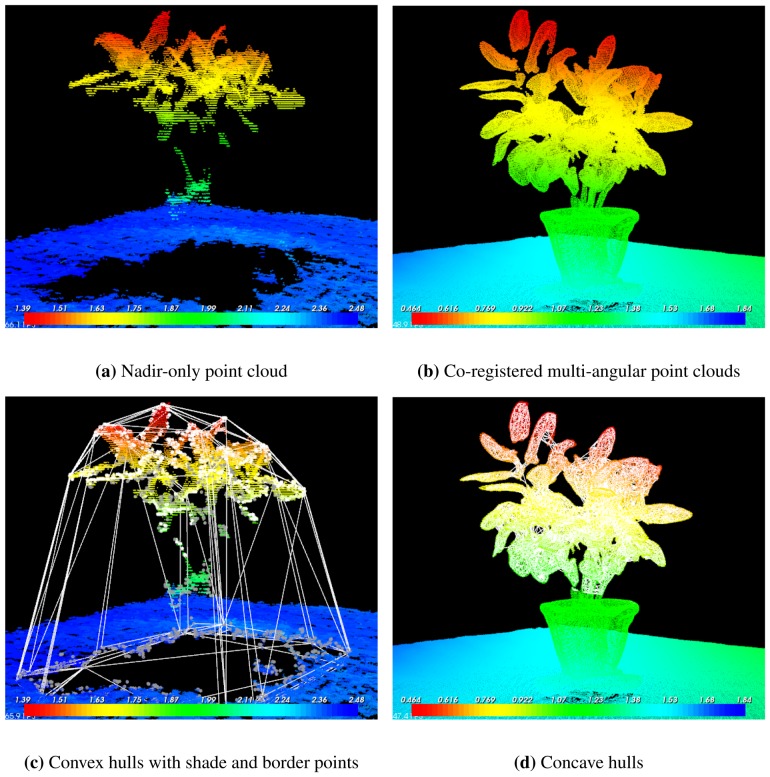
Comparison between nadir-only (a and c; collected using the “Tower Mode”) and co-registered multi-angular (b and d; collected using the “Multi-angular Mode”) point clouds. The upper panels show the point clouds viewed from an arbitrary horizontal perspective. The lower panels show convex (**c**) and concave (**d**) hulls superimposed as white lines. Panel (**c**) shows the shade (grey) and border (white) points used to define the convex hulls. Point cloud color scale is distance (meters) from the sensor at nadir.

1. Noise reduction and filtering to remove outliers: spurious individual points (outliers) were removed using statistical filters. The point clouds were then down-sampled to a 1 cm grid to reduce computational demand.
2. Delineation of individual plants and point extraction: single plants were initially isolated by visually determining the “box coordinates”, *i.e.*, the *x*, *y*, *z* ranges occupied by a plant. Alternative methods for unsupervised plant detection were explored, including planar segmentation of the soil plane and Euclidean clustering. Unfortunately, these methods gave inconsistent results, especially when applied to point clouds acquired in the field (as in Section 2.4). Irregularity of the understory and point clouds that included multiple plants presented particular challenges.
3. Calculation of plant *x*, *y*, and *z* size: each point (*P*) is represented by *x*, *y*, *z* coordinates; plant size along each axes was calculated as *d_i_* = *max*(*P_i_*) − *min*(*P_i_*), where *i* can be *x*, *y*, or *z*, and *P* belongs to the isolated plant points subset.
4. Calculation of plant volume: there are several geometrical algorithms that can be used to calculate the volume of a vegetation point cloud. In principle, these methods should provide a much more accurate estimate of volume than would be possible with a solid shape approximation. Convex and concave hulls have proven especially well suited for this problem e.g., [13]. The convex (concave) hulls *C* of a set of points *Q* = {*p*_1_,*p*_2_,…,*p_n_*} on a plane is “*the unique convex (concave) polygon whose vertices are points from Q and that contains all points of Q*” [24]. This definition can be extended to 3-dimensions, with *C* being a surface mesh instead of a polygon. The volume and structure of a plant should ideally be estimated using concave rather than convex hulls, since this will exclude the empty spaces between branches and leaves. Concave hulls estimation requires a complete cloud of each plant, and it can be applied only to co-registered, multi-angular point clouds. Hulls estimation from nadir-only point clouds can be problematic, because leaves and stems shade each other along the *z* axis, resulting in an incomplete representation of the plant between the larger parts of the crown and the soil. Convex hulls in nadir-only point clouds were estimated after the base points of the plant were determined. We used the plant's shade to define its base. Shade points were identified using a border recognition method based on depth values. This method returns three subsets of points corresponding to borders (shown in white in Figure 2(c)), shades (shown in grey in Figure 2(c)), and veil points.

### 2.3. Preliminary Tests on Kinect

We tested Kinect under a range of conditions to: (a) evaluate the effects of light conditions on the measurements, and (b) compare the information obtained by a fixed-angle looking Kinect (*i.e.*, clouds using the “Tower Mode”) with that obtained by co-registered acquisition (*i.e.*, clouds using the “Multi-angular Mode”).

We carried out these tests on a potted rubber fig tree (Ficus elastica), which was about 1.1 m tall (Figure 1(a)). Ficus elastica's large, smooth leaves are particularly easy to detect using Kinect. The plant was placed on a smooth concrete surface that had an infrared reflectance that differed markedly from that of the plant's leaves. Images were acquired using the “Tower Mode” configuration at 30 minutes intervals beginning 1.5 hours before sunset and ending 1.5 hours after sunset. Additionally, the sensor was detached from the tower and used in “Multi-angular Mode” at night to obtain complete point clouds of the plant.

### 2.4. Testing Kinect in the Field

We also evaluated Kinect's performance and limitations in a more natural grassland setting. A complete assessment of Kinect's reliability in providing canopy structure information in the field might make use of a direct comparison with corresponding ground-based LIDAR measurements. Unfortunately, we lacked access to such a device and consequently compared Kinect's results with ruler-based measurements of plant size. Measuring plant dimensions using rulers is a common practice in field ecology, though the resulting data may inadequately account for a plant's complex shape. Our field comparison was not intended to quantify the absolute accuracy of the plant dimensions measured with the Kinect. In fact, it is likely the Kinect observations are far more accurate than those made with a ruler.

The field site was located on the University of California, Irvine campus (coordinates: 33.640102°*N*, 117.845314°*W*). The area is characterized by a Mediterranean climate; most of the precipitation falls in winter and the summer is reliably dry. The study was conducted in early May, 2011, which was at the end of the growing season. The site was a mosaic of annual grassland and small shrubs, though the grasses had fully senesced at the time of the study. Two large plant species were abundant and reaching the peak of their growing season during our study: Cynara cardunculus (wild artichoke; Figure 1(b,c)) and Picris echioides (bristly ox-tongue). Because of their abundance, their ranges of size, and the contrast the plants presented with the surrounding dry vegetation, these two species were selected for investigation.

Eight plants of Cynara cardunculus and four plants of Picris echioides that ranged in size were selected and marked. We acquired several point clouds for each study plant at night, when the illumination conditions minimized the measurement error. All point clouds acquisitions were done using the “Tower Mode”, with the tower carried by hand to each selected plant. We subsequently measured the basal diameter and height of each plant using a ruler. Both species had a conic shape, and their basal diameters were measured along two orthogonal axes at ground level (one of the two being the maximum diameter, see Figure 1(b,c)). The two basal measurements were subsequently averaged for analysis and comparison. Plant volume was calculated using the manual measurements of basal diameter (*d*) and height (*h*) and commonly used solid shapes including a rectangular box (*V* = *d*^2^ · *h*), cylinder (*V* = *d*^2^ · π · *h*/4), cone *(V* = *d*^2^ · π · *h*/12), parabolic cone (*V* = *d*^2^ · π · *h*/8), and elliptical cone (*V* = *d*^2^ · π · *h*/6). We subsequently harvested the plants and measured their dry biomass.

## 3. Results

### 3.1. Preliminary Tests Using Kinect

**Figure 3.**
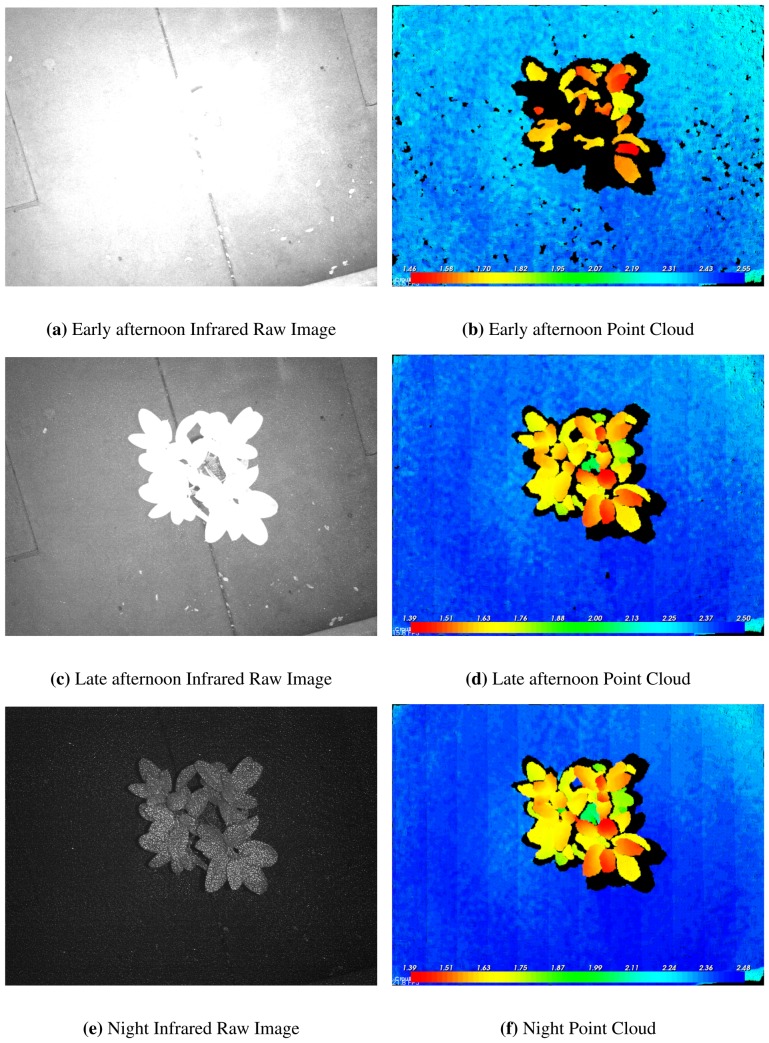
Comparison between raw infrared output from Kinect (**a, c, e**) and corresponding point clouds (**b, d, f**) in different light conditions. All images are for a potted rubber tree plant that was about 1.10 m tall (*i.e*., Figure 1(a)). Point cloud color scale is distance (meters) from the sensor at nadir.

Our preliminary tests showed that light condition and target reflectance influence the depth measurement quality. The series of images in Figure 3 show how depth range increases progressively with darker light conditions. The poor performance obtained under sunlight (see Figure 3(a,b)) was apparently due to the high IR reflectivity of foliage. The reflected IR radiation from sunlight apparently reduced the contrast of the Kinect's laser pattern over vegetated surfaces (Figure 3(a)), resulting in point clouds with many missing points (Figure 3(b)). Nocturnal measurements were needed to obtain well-defined point clouds of the rubber fig plant (Figure 3(e,f)). The background concrete surface, which is the furthest target from the sensor, was detected under all light conditions, though with some missing points in brighter sunlight. The concrete is less reflective in the IR (Figure 3(a,c)), which reduces errors and data gaps caused by sunlight contamination.

Co-registration of multi-angular point clouds gave excellent results in our preliminary test (Figure 2(a,b)). Co-registered point clouds provided a complete representation of the study plant, allowing detailed modeling of the canopy structure using concave hulls (compare Figure 2(d) and Figure 2(c)). Additional tests (not shown in this paper) showed that multi-angular acquisition on plants with smaller-leaves plants and in locations with a complex understory produced less consistent results.

### 3.2. Testing Kinect in the Field

**Figure 4.**
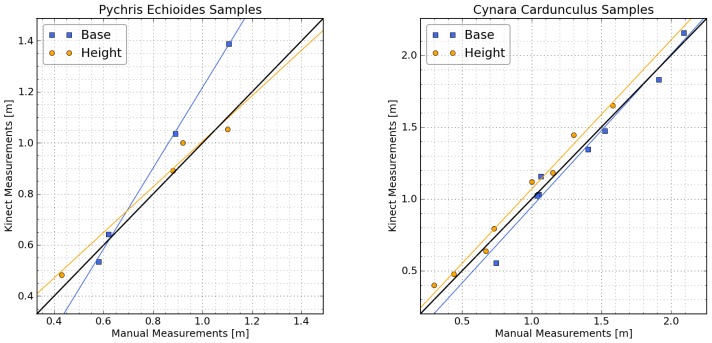
Direct comparison of manual and Kinect measurements of plant basal diameter (“Base”; the average of x and y measurements) and plant height (“Height”). Solid lines represent least squares linear fits.

Comparisons of manual and Kinect-derived measurements of vertical and horizontal plant dimensions are shown in Figure 4. Results showed good agreement for all measurements, especially those on Cynara cardunculus. The Normalized Root-Mean-Squared Error calculated from the identity line (*NRMSE_y_*_=_*_x_*) ranged from 2.7% to 19.1%, with the lowest error corresponding to Cynara cardunculus base measurements and the highest corresponding to Picris echioides base measurements. The stronger agreement obtained for Cynara cardunculus probably reflects the better defined shape of these plants, which simplified the manual measurements.

**Figure 5.**
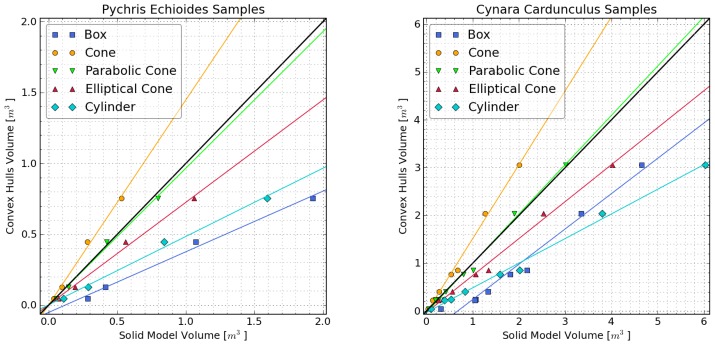
Direct comparison of volumes obtained from convex hulls and those derived from manual measurements with various solid shape approximations. Solid lines represent least squares linear fits.

Plant volume estimated from the manual field measurements using box, cylinder, cone, parabolic cone, and elliptical cone solid models was compared with that obtained from the Kinect using convex hulls (Figure 5). *RMSE_y_*_=_*_x_* values ranged from 1.0% to 106.1%. The elliptical cone provided the best shape approximation for both plant species, with a *RMSE_y_*_=_*_x_* of 1.0% for Cynara and 2.1% for Pychris. Box and cylinder models, which are frequently used for canopy modeling, had the largest *RMSE_y_*_=_*_x_* values (from 57% to 106%).

**Figure 6.**
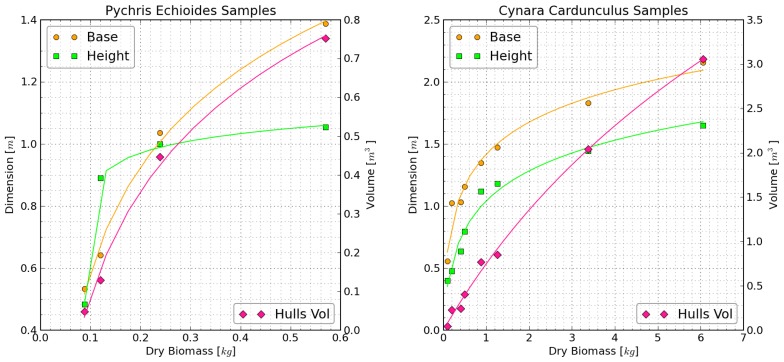
Allometric relations between Kinect-derived dimensions (base and height) and dry biomass measurements. Solid lines represent least squares logarithmic fits of the form *y* = *A* + *B* · log(*x* + *C*)/(*log*(*D*) + 1).

Dry biomass was compared with the Kinect-determined heights, diameters, and volumes to derive allometric relationships (see Figure 6). The various measures of plant size were well correlated with plant biomass through a logarithmic function of the form: *y* = *A* + *B* · log(*x* + *C*)/(*log*(*D*) + 1). The resulting coefficients of determination (*r*^2^) ranged from 0.97 to 1.0.

## 4. Discussion

### 4.1. Strengths and Limitations of Kinect Sensors

The Microsoft Kinect sensor combined with the Point Cloud Library provided measurements of plant height and base diameter that agreed well with the corresponding manual measurements. Similarly, the three-dimensional convex hulls estimations provided useful information on plant volume and shape, which are relevant to biophysical canopy modeling and biomass estimation. The structural information retrieved with Kinect was used to obtain allometric relations for two species of plants. The light weight, small size, fast acquisition time, low power requirement, and cost make the Kinect a promising tool for rapid field surveys of canopy structure, especially in small-statured vegetation.

It is important to acknowledge the Kinect sensor's limitations for vegetation sensing. The Kinect was unable to measure vegetation in daylight, and the measurement range was limited to a few meters. Nighttime measurements may be difficult in field sites with limited accessibility. Measurements from a fixed location, such as the top of a flux tower, circumvent this problem, but would be limited spatially by the Kinect's measurement range.

The results for images collected in “Tower Mode” were less consistent in situations with densely packed vegetation or with complex, live ground cover. The “Multi-angular Mode” is particularly promising and allows the creation of point clouds with remarkable detail (Figure 2(d)), but smaller leaves and dense vegetation can create problems during the co-registration process.

### 4.2. Perspectives for the Future

The Kinect's limitations for measuring vegetation in the field are not surprising; the Kinect was designed for video-gaming indoors rather than ecologic observations in the field. Nonetheless, the Kinect is already useful for some ecological applications in its present forms, and the measurement principle opens possibilities for designing new sensors dedicated to vegetation characterization.

New vegetation sensors based on the Kinect's design could use a more powerful laser source to increase the measurement range and allow characterization of taller vegetation. This might also improve the daylight observations, especially if the projector's wavelength could be selected to enhance the contrast over vegetated targets within sunlit environments. The development of a depth sensor for daylight use would allow retrieval of images from the aligned RGB camera; the RGB information associated with each point could then be used for phenological studies e.g., [25].

Alternatively, the Kinect approach might be used to determine plant spectral reflectance along with the 3-dimensional structure. The intensity of the laser source is known, and reflectance can be calculated once the intensity of the reflected speckled pattern is retrieved at each frame. In principle, multiple illumination wavelengths could be utilized, allowing the calculation of spectral vegetation indices. The use of active rather than passive illumination for multi-spectral reflectance measurements in the field has been tested in recent studies e.g., [26,27]. Active illumination has the advantage of eliminating dependence on weather or light conditions. Moreover, the source-sensor geometry is fixed and known, avoiding the need to consider the Bidirectional Reflectance Distribution Function (BRDF) [26], though discrete laser returns still depend on factors such as vegetation structure, geometry, and angles of incidence.

Improvements could be made to the acquisition procedure, the software and the data processing. Algorithms for acquisition and co-registration of multi-angular point clouds could be improved to allow acquisition in the field, where small plants or complex understory currently create errors. Multi-angular measurements in the field may be readily acquired by hand during field campaigns, or using pan-tilt units (e.g., on flux towers), or with multiple sensors, or with sensors flown at low altitude e.g., [28–30]. More accurate algorithms for quantifying structural parameters such as Leaf Area Index and Leaf Angle Distribution could be developed starting from co-registered point clouds.

## 5. Conclusions

The Microsoft Kinect shows potential for measuring the structure of small plants in the field, but its design currently limits the range of possible applications for ecological studies. Nevertheless, the development of software and inexpensive consumer electronics is opening exciting possibilities for ecological applications. Further research on these technologies is inevitable given the rapid development of inexpensive and easy-to-use sensors. This, in turn, can increase the spatial and temporal coverage and availability of ground data, providing better information for validation of remote sensing measurements and improving biophysical canopy modeling (e.g., Figure 2(d)).
